# Science publishing 2035

**DOI:** 10.1098/rsnr.2016.0033

**Published:** 2016-08-24

**Authors:** Rebekah Higgitt

**Affiliations:** School of History, Rutherford College, University of Kent, Canterbury, Kent CT2 7NX, UK

It is clear that we cannot disentangle the question of what the scientific journal will look like in twenty years' time from the question of what the university and academia will look like. The pressures of competitive, individual research assessment, within an increasingly marketized world of higher education, would indicate a future of science publishing that is more closed, more niche, more expensive and more disconnected. We may find, for example, if universities compete for their niche by specializing in certain fields, or in teaching over research, that some journal subscriptions are beyond their means or scope. This would mean that academics as well as other interested publics could lose access to new research. An alternative vision follows the open access models of the science journal *PLoS ONE* or the non-profit publisher Open Library of the Humanities. Here content is open to all, and the process of peer review can be made visible and invite a wide range of commenters. In addition, their flexible formats can help us to develop ways to present research in connected, dynamic and updatable ways. While academics will probably continue to maintain a mixed economy, it must be acknowledged that this will be differently accessible to academics depending on field, institution, career stage and individual security and productivity. Now is the time to decide if we would prefer a less divisive future, and to understand that the open model requires action and change.

Such change requires us to find different ways of assessing what counts in academic careers and of rewarding activities that work against the individualistic tendencies and atomization of modern academia. This includes encouraging open publishing, self-publishing, work that has an impact on colleagues outside particular fields or the wider public, sharing our colleagues' research as well as our own and providing a supportive environment for scholars and scholarship. Such an approach has been described as ‘slow scholarship’, which may involve publishing less, or publishing differently, but is designed to resist division and the move to define careers by publication metrics. It also, crucially, should encourage care and diversity, and has thus been taken up by some as a feminist initiative. It requires imagining a situation very different from that in which most of us find ourselves today. This is where history, including the history of scientific publishing, can be an important tool: it shows that things have been different in the past and indicates that they will change again in the future in ways that we may be able to shape. Historical awareness can help us to notice and reflect on the situation in which we find ourselves, and encourage us to question it and the structures behind it, as well as equipping us to unpick assumptions about what successful science and scholarship ‘should’ look like. Scientific journals have proved to be malleable entities and by exploring what has made and changed them we can think comparatively and, perhaps, ‘outside the box’.

